# Phytate (Inositol Hexakisphosphate) in Soil and Phosphate Acquisition from Inositol Phosphates by Higher Plants. A Review

**DOI:** 10.3390/plants4020253

**Published:** 2015-05-22

**Authors:** Jörg Gerke

**Keywords:** Inositol hexakisphosphate (IHP), phytate, IHP sorption, phosphatases, humic-Al(Fe)-IHP complexes, carboxylate mobilization

## Abstract

Phosphate (P) fixation to the soil solid phase is considered to be important for P availability and is often attributed to the strong binding of orthophosphate anion species. However, the fixation and subsequent immobilization of inositolhexa and pentaphosphate isomers (phytate) in soil is often much stronger than that of the orthosphate anion species. The result is that phytate is a main organic P form in soil and the dominating form of identifiable organic P. The reasons for the accumulation are not fully clear. Two hypothesis can be found in the literature in the last 20 years, the low activity of phytase (phosphatases) in soil, which makes phytate P unavailable to the plant roots, and, on the other hand, the strong binding of phytate to the soil solid phase with its consequent stabilization and accumulation in soil. The hypothesis that low phytase activity is responsible for phytate accumulation led to the development of genetically modified plant genotypes with a higher expression of phytase activity at the root surface and research on the effect of a higher phytate activity on P acquisition. Obviously, this hypothesis has a basic assumption, that the phytate mobility in soil is not the limiting step for P acquisition of higher plants from soil phytate. This assumption is, however, not justified considering the results on the sorption, immobilization and fixation of phytate to the soil solid phase reported in the last two decades. Phytate is strongly bound, and the P sorption maximum and probably the sorption strength of phytate P to the soil solid phase is much higher, compared to that of orthophosphate P. Mobilization of phytate seems to be a promising step to make it available to the plant roots. The excretion of organic acid anions, citrate and to a lesser extend oxalate, seems to be an important way to make phytate P available to the plants. Phytase activity at the root surface seems not be the limiting step in P acquisition from phytate. Phytate is not only bound to inorganic surfaces in soil but can also be bound, similar to orthophosphate, to humic surfaces via Fe or Al bridges. Humic-metal-phytate complexes may be transported in the soil solution to the roots where hydrolysis and uptake of the liberated P may occur. Research on this topic is strongly required.

## 1. Introduction

Myoinositolhexakisphosphate (phytate) is the quantitative most important inositol phosphate found in soil. Other stereoisomers and lower phosphorylated species are also found [1,2,3].

Phytate is a phosphorus storage molecule and constituent in cereals and grains [4] and is introduced to the soil by various plant residues, and by animal manure [5,6,7]. Phytate represents an immense reservoir. Fifty-one million tons of phytate is found every year in commercially produced fruits and crop seeds [8]. According to [4], this phosphate quantity is equivalent to about 67% of the annual worldwide phosphate (P) application in mineral fertilizers, indicating the quantitative importance of phytate P for the P cycle in soil. Phytate is strongly bound to the soil solid phase [9,10,11] and significantly contributes to the pool of organic soil phosphate (P_o_). P_o_ can account for between almost nil and 95% of the total soil phosphate (P_t_) [12,13,14,15,16,17]. Besides phytate, other inositol phosphate isomers are found in soil, mostly highly phosphorylated inositol penta*kis* and hexa*kis* phosphates. Lower phosphorylated inositol phosphates seem to be of minor quantitative importance in soil. In the following paper we will term the highly phosphorylated inositol molecules inositol hexa*kis*phosphates (IHP), taking in mind that phytate is the dominating molecule within this group and inositol penta*kis*phosphate isomers may also be present. IHP are often the most important identifiable soil P_o_-fraction [18]. However, the fraction of “unknown” P_o_ is often the dominant fraction in soil, often consisting of high molecular mass organic matter P, mainly associated with humic substances in soil [19]. The unknown P_o_ fraction in soil is often forgotten in the discussion of P_o_-P acquisition by higher plants. IHP itself is often associated with high molecular mass humic substances [20,21,22,23,24,25,26,27,28].

Phosphate is an essential macronutrient for higher plants. Worldwide, the P reserves are strongly limited and peak P (similar definition to peak oil) had been three decades ago [29,30]. Phosphate is strongly bound to the soil solid phase and is taken up by the plant roots as H_2_PO_4_^−^ or HPO_4_^2−^ ion (P_i_), the soil solution being replenished from the soil solid phase. *Thus, to acquire inositol P*, *IHP must be dissolved in the soil solution and the ester bond must be split.* However, the mechanisms by which IHP accumulate in soil and by which way phytate P can be acquired by the plant roots is not fully understood.

This review aims to give an overview on the main processes and the gaps of knowledge on IHP accumulation in soil and IHP-P acquisition by the plants.

## 2. The Binding of Inositol Hexa*kis*phosphate in Soil

The input of IHP to soils is quantitatively important. After entering the soil, IHP is strongly bound to the soil solid phase. The comparison of IHP and P_i_ bonding in soil may be useful. The orthophosphate anion (P_i_) itself is strongly bound to the soil solid phase, mainly to Fe-OH- or Al-OH-groups [27] (p.11). Soils with a high concentration of reactive Fe/Al-OH groups strongly bind P_i_. In soil chemistry, such groups can be determined by the extraction with acid oxalate solution, Tamms reagent [31]. Binding sites in soil are centered on iron oxides such as goethite or ferrihydrite, Al-oxides, such as boehmite or gibbsite or edges of clay minerals. The mechanism of bonding is surface complexation, by which OH or OH_2_ groups of the adsorbent are replaced by the P_i_ anion [27] (pp. 10,11). Several surface complexation models to describe phosphate adsorption have been developed and successfully applied to P_i_ adsorption by soil components and soils [32,33,34]. IHP is also firmly bound to soil components and to the soil solid phase [10,11,19]. McKercher and Anderson [9] showed that IHP sorption to soil solid is stronger than that of the P_i_ anion. In the same paper, they also showed that even inositol tri*kis*phosphate is stronger sorbed than the orthophosphate anion, whereas inositol mono*kis*phosphate sorption was lower compared to the P_i_ anion. The higher the degree of phosphorylation of the inositol ring, the higher the sorption of the inositol phosphate to the soil solid phase. For goethite and ferrihydrite, Celi and Barberis [10] showed that the sorption of IHP-P was higher than that of the P_i_ anion. They interpreted their results on the IHP adsorption to the Fe-oxide by assuming that four phosphate groups contributed to the binding to goethite and two phosphate groups to the binding to ferrihydrite on the basis of the adsorption maximum determined with the aid of the Langmuir-isotherm.

Yan *et al.* [35] investigated the sorption of phytate to amorphous Al-oxide by spectroscopic methods and found that phytate binding induced the formation of Al-phytate precipitates at the oxide surface.

Often, a great proportion of inositol phosphates is found in the high molecular mass fraction associated with humic substances [20,21,22,23,24,25,26]. The bonding of inositol phosphates to soil humic substances is often very strong. The reaction of the humics with 1 M NaOH did not liberate inositol or inositol phosphates [21], but hydrolysis with 6 M HCl at 100 °C released inositol from the humics [23], indicating a very strong incorporation of inositol and probably inositol phosphates into the humic matrix.

Other phosphate esters, such as RNA, DNA or sugar phosphates, react only moderately with the soil solid phase, which may be the reason for their low contribution to soil organic phosphate [15,16,17,18]. The enzymatic cleavage of the inositol-phosphate ester bond and the mineralization of the inositol ring may be reduced by surface complexation to the soil solid. Thus, the extend and strength of sorption of P_o_ to the soil solid phase and its persistence and accumulation in soil may be closely related and may be the reason for the accumulation of IHP in soil compared to other P esters in soil.

Another tool, ^31^P-nuclear magnetic resonance (NMR) spectroscopy of soil extracts gives further information on soil P_o_ and IHP in soil [36]. By the aid of this spectroscopic method, the discrimination between orthophosphate, phosphate monoesters, phosphate diesters and phosphonates in the extracting solution is achieved. Phosphate monoesters mainly consist of inositol phosphate, with IHP probably the dominating form [3,36,37].

Two main limitations of ^31^P-NMR spectroscopy should be mentioned. First P_i_ and P_o_ extractability from soil by various extracting solutions is limited. For example, Turner *et al.* [38] extracted 18 semiarid arable soils by a mixture of 0.25 M NaOH +50 mM EDTA solution and investigated the extracts by means of ^31^P-NMR spectroscopy. The extractability of total P by this relatively effective extracting solution was between only 12% and 45%, with a medium value of 26.4%.

With the same extracting solution, Turner [39] extracted P from three soils the extractability of P_o_ being between 20% and 30% compared to the determination of P_o_ by the ignition method. Low relative amounts in the extracts for solution ^31^P-NMR spectroscopy limit the interpretations of the results. The results of Turner *et al.* [3], who found for three soils a P recovery of more than 90% in the NaOH/EDTA extracts, are probably an exception. Low recovery of P_t_ and P_o_ in soil extracts makes it impossible to quantitatively determine the content of IHP in soil by ^31^P-NMR spectroscopy of soil extracts even under the assumption that most of the monoesters are IHP.

Nevertheless, Turner [18] reported results on the content of inositol hexakisphosphate from own and other investigations to be between 1 and 460 [mg·P/kg soil].

Solid phase ^31^P-NMR spectroscopy may overcome the problems of low P recovery of the extracting solutions. This method is too insensitive with a relatively low signal to noise ratio [36].

The second limitation of ^31^P-NMR spectroscopy of soil extracts lies in the fact that, for IHP, but also other P_o_ forms in soil, the spectra give no information on the bonding of the different P_o_ molecules especially of the bonding to soil humic substances. However, inositol phosphates are firmly bound within the humic matrix and may chemically behave very different in soil compared e.g., to free IHP [21,23]. The main bonding mechanism of IHP is probably that via Fe(III)- or Al-bridges to the humic matrix, similar to the binding of the orthophosphate anion [19,27,28,40].

**Figure 1:**
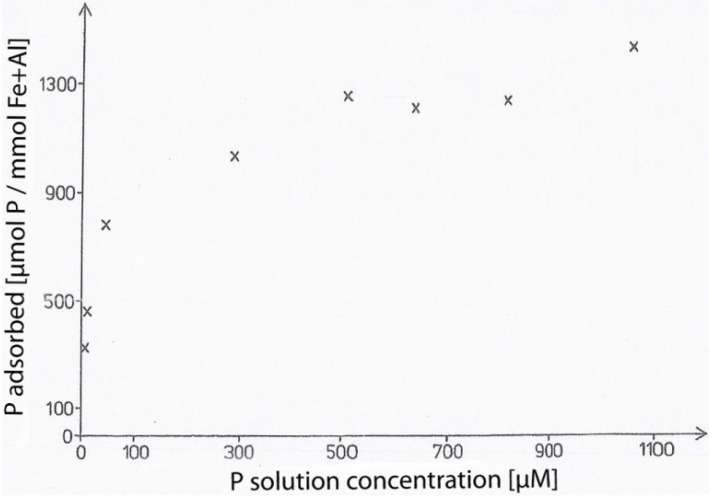
Adsorption of *myo*-inositol hexa*kis*phosphate to native humic-Al(Fe) complexes at pH 6.2 in 0.1 M NaCl from Gerke [27] (p. 208).

Gerke [27] (p. 280) measured the adsorption of phytate to humic-Fe(Al) complexes in 0.1 M NaCl at pH 6.2 (Figure 1).

The adsorption exceeded 1300 [μmol phytate-P/mmol Fe+Al], indicating that Fe and Al complexed by the humics bind phytate, which is in agreement with Negrin *et al.* [28]. Further research (Gerke, unpublished) on the binding of phytate to model humic substances nearly free from Fe and Al showed that phytate was not bound in the system at pH 6.2 in 0.1 M NaCl, a result similar to that of the binding of the orthophosphate anion [41]. Figure 1 shows that native Fe and Al complexed by soil humic substances exhibit a high capacity of phytate sorption.

Speculations on the covalent IHP bonding to the humic surface during adsorption studies as well as on hydrogen bonding by Celi and Barberis [11] are not supported and ignore bonding via Fe(Al)-bridges, which is likely the most important mechanism. IHP in soil, incorporated into the matrix of humic substances, will behave different from IHP in the soil solution or bound to inorganic surfaces such as Fe- or Al-oxides.

Preparation procedures for the extraction, purification and dissolution of soil humic substances often include purification steps with relatively strong acid or cation exchanger to remove most of the inorganic “impurities”. But these impurities of humic substances, Fe(III) or Al(III) or sometimes Cu(II) are decisive for the binding of P_i_ or IHP to the humic surface.

The acquisition of P_i_ from soil by the plant roots requires the desorption/dissolution of the orthophosphate anion from the soil solid phase and its diffusion in the soil solution to the plant roots (for review see e.g., [42,43]). Similar processes are expected for the IHP desorption and its transport to the roots. However, IHP is much stronger bound than P_i_, the equilibrium concentration of free IHP species in the soil solution being expected to be very low. IHP bound to the soil humic substances may also be dissolved if the associated humic molecule is dissolved into the soil solution, which may increase the solubility of IHP in soil.

In summary, IHP in soil accounts for an important proportion of P_t_ in many soils, sometimes it is dominant. The reason for the IHP accumulation may at least be partly explained by the strong bonding of IHP to the soil solid phase, which may hinder the microbial degradation. The result of McKercher and Anderson [9] also demonstrated that also inositol tri*kis*phosphate is stronger sorbed to soil than the orthophosphate anion and only the monophosphorylated inositol showed a weaker sorption to the soil solid compared to the orthophosphate anion. We conclude that with a higher degree of phosphorylation of the inositol ring, a higher sorption to the soil solid phase and a stronger persistence in soil is achieved.

The accumulation of IHP and possibly inositol penta*kis*phosphate in soil is strong evidence for the relatively low contribution to P acquisition by plants from this source.

Two principal hypothesis on the limiting step of P acquisition from soil-IHP can be formulated, (I) A low phosphatase activity in soil or at the root surface by which the rate of release of the P_i_ anion from IHP is the limiting step in P acquisition; and (II) the low rate of IHP desorption from the soil solid phase, which limit the IHP equilibrium concentration in the soil solution, and consequently the diffusion of IHP-P to the roots and its P acquisition by higher plants.

## 3. The Role of Phosphatases (Phytases) in IHP-P Acquisition from Soil by Plants

Roots of higher plants absorb P from solution as orthophosphate anion. In the pH range of most soils, the principle phosphate species in the soil solution, which are taken up by the roots, are H_2_PO_4_^−^ and HPO_4_^2−^ [44,45]. Phosphate esters such as phytate are not directly taken up by the roots, the cleavage of the phosphate anion from the inositol ring is a required step, which is catalyzed by phosphatases.

Conflicting results exist whether, and under which conditions, phytate and IHP in general can be utilized as a P source by the roots.

To discriminate the effect of IHP sorption and the effect of phosphohydrolases in experiments, sometimes plant cultivation in non-IHP sorbing substrates, such as quartz sand, nutrient culture or agar techniques are used. The reported results on the role of phosphatases in P acquisition from IHP are very contrasting.

Tarafdar and Claassen [46] measured the P acquisition in aseptic nutrient solution by *Trifolium alexandrinum* from inorganic P, glycero phosphate, lecithin and phytate and found a similar P uptake from these different P forms and a similar hydrolysis of the different P_o_ forms. In this study, hydrolysis of phytate was not the limiting step in P acquisition in solution. In a strong P deficient soil, Tarafdar and Claassen [46] also found a similar plant P uptake from P_i_, glycero phosphate, lecithin and phytate. They concluded that also in this soil, P hydrolysis by phosphatases was not the limiting step in P acquisition from phytate.

Nearly opposite results were found by the group of Richardson and coworkers [47,48]. Hayes *et al.* [47] compared the P acquisition by different pasture species from agar culture supplied with orthophosphate, glucose-1-phophate, or IHP. Compared to the other two P sources, they found a poor P availability of IHP-P. Adding phosphatases strongly improved P acquisition from IHP. The authors concluded that even under conditions of high P solubility, the enzymatic hydrolysis to be the limiting step in P acquisition from IHP. The results of Findenegg and Nelemans [49] on the acquisition of phytate from quartz sand culture seem to support the work of Hayes *et al.* [47]. The differences in the papers of Tarafdar and Claassen [46] and Findenegg and Nelemans [49] on the phytate P acquisition from soil may be caused by differences in the soils that were used. However, the differences in phytate P acquisition from “non-sorbing” substrates, agar, nutrient solutions or quartz sand may be due to unintended phytate sorption. Sorption in soil chemistry includes adsorption to the soil solid phase, surface precipitation or precipitation, *i.e.*, chemical processes that reduce the concentration of a definite chemical species in the soil solution. The Ca-concentrations of the phytate containing substrate were 0.45 mM [46] or 4.0 mM [47,49]. In the latter two papers, Ca-phytate precipitates or suspended precipitates may have reduced the phytate availability and may seriously question the hypothesis of limited activity of phosphatase. In the experiments of Tarafdar and Claassen [46], phytate sorption is less probable, whereas in that of Hayes *et al.* [47] and Findenegg and Nelemans [49], the relatively high Ca-concentration of the substrate makes the existence of Ca-phytate precipitates probable.

This interpretation is supported by the results of Lung and Lim [50] and Beißner [51]. Lung and Lim [50] showed for tobacco plants an adaptive increase in phytase activity during P starvation accounting for more than 18% of the acid phosphatase activity in P starving plants. Tobacco plants grew better if supplied with Mg-phytate compared to Ca-phytate. Lung and Lim [50] concluded from their results that the solubility of phytate and not the restricted hydrolysis of the ester bond is the limiting factor in phytate P acquisition in soil.

Beißner [51] investigated the P acquisition of sugar beet plants in quartz sand culture from phytate P. He also attributed the limited phytate P acquisition compared to that of P_i_ to the low solubility of phytate even in the quartz sand system, probably due to the precipitation of Ca-phytate.

George *et al.* [48] used the agrar technique of Hayes *et al.* [47] and found that several genotypes of *Trifolium subterraneum* with an overexpression of phytase took up more P from phytate compared to the wild types. Again the problem may be suspended or precipitated Ca (Mg)-phytate with a reduced solubility. Under such conditions of a relatively low phytate solubility, a higher phosphatase activity may induce the dissolution of precipitated phytate. However these results cannot be transferred to the situation in soil. The soil solution concentrations of IHP are expected to be extremely low [52], because of the very strong IHP sorption to the soil solid phase [9]. Under such soil conditions, an increase in phosphatase activity in soil has not proven to be effective in P mobilization.

An effect of increased phytase activity on P acquisition from IHP as shown by Hayes *et al.* [47] and George *et al.* [48] is limited to artificial substrates where the free IHP solubility is limited but relatively high compared to soil conditions. If, however, the IHP solubility is not limited as in the experiments of Tarafdar and Claassen [46] and Adams and Pate in sand culture [53], then IHP is a P source that can be used at similar rates compared to other P_i_ or P_o_ sources, again indicating that IHP solubility and not limited phosphatase activity limits P acquisition from IHP.

## 4. Mobilization of IHP from the Soil Solid Phase: Mechanisms and Relevance

The accumulation of IHP (phytate) in soil compared to other P-esters [14,15,16] can be attributed to the stabilization of IHP by the rapid adsorption and surface complexation [28], in some cases formation of ternary IHP-complexes at the soil solid phase [35] and the reaction of IHP with humic substances mainly via Fe(Al)-bridges and incorporation into its structure [19,20,21,23,28].

The immobilization of highly phosphorylated inositol phosphate seems to be similar to that of the orthophosphate anion (P_i_), however immobilization and fixation processes may be much stronger for IHP compared to the P_i_ anion. Mobilization and desorption strategies to make IHP-P plant available are mainly unknown.

The desorption and mobilization of P_i_ may, however, show similar reaction pathways to that of IHP.

In P fixing soils, the P_i_ anion can be mobilized by the excretion of di- and tricarboxylic acid anions mainly by citrate and to some extend by oxalate [54]. These di- and tribasic anions can desorb the P_i_ anion from the soil solid phase by ligand exchange, replacing P_i_ at the soil solid with the carboxylate anion. The organic anions can also dissolve Fe and Al, thereby destroying the sorption sides of P_i_. Moreover, the carboxylate anion can dissolve humic molecules to which, via Fe/Al-bridges, the P_i_ anion is bound. P_i_ is dissolved as humic-Fe(Al)-P complex [19].

Similar reactions may occur during the mobilization of IHP bound to the soil solid, but results are rare. For example, adsorption studies of IHP to humic substances do not exist, with the exception of Gerke [27] (p. 208) (Figure 1). Despite of the emphasis that has been put on the fact that in soil IHP is often associated with the high molecular mass fraction [10,11,18], the experimental foundation of this statement is relatively old [20,21,22,23]; and, there is no actual focus on, probably, the most important reaction mechanism, the chemical interaction between IHP and humic substances via Fe/Al-bridges [19]. Instead, speculations can be found on the mechanism of hydrogen bonding and even covalent bonding between IHP and humic substances [10,11].

In soil, the orthophosphate anion can be mobilized by the root release of citrate or oxalate the by far most efficient carboxylates. By the aid of calculations with a mathematical model originally developed by Nye [55,56] and based on the measured rate of release of carboxylates [27], it was shown that P_i_ mobilization by citrate is strongly important in *red clover*, and to a certain extend in *white clover*, whereas the carboxylate excretion in *ryegrass* is so small, that no P mobilization by this plant species could be found [57].

P_i_ mobilization is extremely important in cluster forming plant species such as white lupin and yellow lupin. Under P deficiency, these plant species form bottle brush like cluster roots with intensive citrate excretion and P mobilization in the cluster root rhizosphere [58,59,60,61]. The P mobilization by citrate and the P_i_ acquisition of the mobilized P is very strong in the cluster root rhizosphere, because of the strong citrate accumulation and because of the efficient uptake of the mobilized P [57].

Adams and Pate [53] investigated the P acquisition by white lupin (*Lupinus albus* L. with cluster roots) and narrow leaf lupin (*Lupinus angustifolius* L. without cluster roots) in quartz sand culture and in P fixing soil from orthophosphate, choline phosphate and phytate. In quartz sand, both plant species took up P_i_ and phytate-P with the same rate, indicating no phytate sorption and no limitation of P acquisition by a reduced phosphatase activity. The results in quartz sand culture are in accordance with those of Tarafdar and Claassen [46] and Lung and Lim [50] and in strong contrast to the results of Hayes *et al.* [47] and George *et al.* [48]. In P fixing soil, the both lupin species differed in P acquisition from phytate. *Narrow leaf lupin* took up P from Pi and glycerol phosphate at similar rate, whereas P uptake from phytate in P fixing soil was very small. *White lupin* also took up P from Pi and glycero-phosphate at similar rate. The P acquisition from phytate was smaller but showed an increasing P uptake with increasing rate of phytate application [57]. The results of Adams and Pate [57] have proven, that in both lupin species phytate P acquisition is limited by the phytate sorption/fixation to the soil solid. Insufficient phosphatase activity was not the limiting factor in P acquisition. The results of Adams and Pate [57] who showed for the first time in which way phytate P can be acquired by plants in P fixing soil, did not receive the adequate attention or were misinterpreted.

For example, Richardson *et al.* [37] interpreted the results as follows: “Adams and Pate (1992) showed…, but in contrast, phytate was only accessed by plants grown in sand…”. With this interpretation, Richardson *et al.* [37] ignored that in sand IHP was as good as P source as orthophosphate for two lupin species (which is contrary to their own hypothesis as formulated by Haynes *et al.* [47] and George *et al.* [48], of phosphatase activity driven P acquisition from phytate in low P sorbing culture) and Richardson *et al.* [37] stated wrongly that phytate P was not acquired by both lupin species in the soil experiment by Adams and Pate [53].

Richardson *et al.* [62] and more recent Giaveno *et al.* [63] and Giles *et al.* [64] repeated the view that phosphatase activity is a limiting step of IHP acquisition. The contrary results of Tarafdar and Claassen [46], Adams and Pate [53], Beißner [51] and Lung and Lim [50] were ignored or misinterpreted.

Both studies, Beißner [51] and Lung and Lim [50], summarized and concluded that the availability of IHP-P, *i.e.*, the buffering is the limiting step in P acquisition from IHP in soil.

## 5. The Role of Soil Microorganisms in the P Acquisition from IHP-P

The possible role of soil microorganisms may include three aspects:

- the role of mycorrhiza;
- the effect of microorganisms on the mineralization on root released carboxylates; and/or
- the role of soil microorganisms in producing phosphatases (phytases).

a. Some evidence exists that arbuscular mycorrhizal symbioses with higher plants uses the same soil P fractions than plants without mycorrhiza [65,66]. If these findings can be generalized, then the hyphae of mycorrhizal fungi mainly have the function of increasing the exploitable soil volume, similar to root hairs (see for a very informative summary on root hairs, Jungk, [67]). An effect of hyphae to increase IHP solubility by the release of mobilizing agents seems to be unimportant.

b. Soil microorganisms may reduce the effect of root-released carboxylates on P mobilization and acquisition by microbial degradation of the carboxylates. The accumulation of carboxylates at the soil solid phase is a required step in P mobilization by carboxylates [54,57]. Gerke [68] recently summarized the effect of carboxylate release and accumulation in the rhizosphere on the acquisition of P_i_ and P_o_. Carboxylates in the soil solution are easily and quickly mineralized by microorganisms, whereas the adsorption to the soil solid phase, e.g., to Al-oxides [69] or Fe-oxides [70] strongly inhibits the degradation, which may be a prerequisite for the effect of carboxylates on P_i_ and IHP acquisition in soil [68].

c. In soil, many fungal or bacterial communities produce phosphatases and hydrolyze ester-P [71]. Furthermore, microbial P account for around 10% of total P in grassland and for around 5% in arable soil [72]. However, the role of microorganisms for the P acquisition of higher plants from P_o_ in general and IHP-P is unclear. Quiquampoix and Moussain [73] reviewed the role of phosphatases in soil for the hydrolysis of P_o_. However, the close relation between the phosphatase activity in the rhizosphere, the hydrolysis of organic P and the P acquisition as shown by Tarafdar and Jungk [74] and Chen *et al.* [75] does not answer the question of the origin of the enzymes, plant or soil microbes. Recent results by Belinque *et al.* [76], however, show that phytate-P acquisition by oil seed rape, sunflower and soybean in nutrient solution is not improved by microbial inoculation indicating that plant-derived phosphatases hydrolyze phytate with such a high rate, that P acquisition from phytate is similar to that from P_i_. The results of Belinque *et al.* [76] are similar to that of Tarafdar and Claassen [46] and may be interpreted that the phosphatase activity of higher plants at the root surface may satisfy the demand to hydrolyze Po transported to the root surface via diffusion in the soil solution. Microbial phosphatase activity may, however, be important for the microbial P turnover in soil [72].

## 6. Summary and Outlook

Various phosphate esters are entering the soil. Their accumulation is related to strength of bonding to the soil solid phase. This is recognized to be the reason for the accumulation of the highly phosphorylated inositol phosphates in soil, mainly inositol hexa*kis*phosphate.

IHP accounts for between nil and about 45% of the P_o_ in soil. In many soils most of the P_o_ is attributed to “unknown P_o_ pool”.

Often, the association between IHP and soil humic substances is important, thus most of the inositol phosphate in soil is in the high molecular mass fraction. The knowledge on the reactions and P availability of high molecular mass associated IHP is small.

The accumulation of IHP in soil is an indicator of low P availability of IHP-P to the plants.

The acquisition of IHP-P may be strongly improved by root exudates, which may increase the solubility of IHP in soil. The excretion of citrate and oxalate by plant roots may be promising to improve IHP-solubility in soil and its P acquisition by the roots. Experimental studies on this topics are urgently needed.

Two main future research goals considering IHP in soil and its P acquisition may be emphasized:

1. Research on the binding of IHP in soil with a main focus on the binding to the soil humic substances.
2. Mobilization of IHP and other inositol phosphates from the soil solid phase by root exudates with the central focus on the role of di- and tricarboxylic acids and the acquisition of IHP-P by the roots of higher plants.

Results on the IHP binding in soils and its mobilization probably mainly by di-and tricarboxylic acids may be incorporated into mathematical models on the acquisition of mobilized IHP-P by plant roots, as has been successfully applied for the acquisition of inorganic P [54,57].
